# Differential Expression of Several miRNAs and the Host Genes *AATK* and *DNM2* in Leukocytes of Sporadic ALS Patients

**DOI:** 10.3389/fnmol.2018.00106

**Published:** 2018-04-04

**Authors:** Katarina Vrabec, Emanuela Boštjančič, Blaž Koritnik, Lea Leonardis, Leja Dolenc Grošelj, Janez Zidar, Boris Rogelj, Damjan Glavač, Metka Ravnik-Glavač

**Affiliations:** ^1^Department of Molecular Genetics, Faculty of Medicine, Institute of Pathology, University of Ljubljana, Ljubljana, Slovenia; ^2^Division of Neurology, Institute of Clinical Neurophysiology, University Medical Centre Ljubljana, Ljubljana, Slovenia; ^3^Department of Biotechnology, Jožef Štefan Institute, Ljubljana, Slovenia; ^4^Biomedical Research Institute, Ljubljana, Slovenia; ^5^Faculty of Medicine, Institute of Biochemistry, University of Ljubljana, Ljubljana, Slovenia

**Keywords:** amyotrophic lateral sclerosis, sporadic ALS, leukocytes, differential expression of miRNAs, down-regulation of *AATK*, up-regulation of *DNM2*, Slovenian population

## Abstract

Genetic studies have managed to explain many cases of familial amyotrophic lateral sclerosis (ALS) through mutations in several genes. However, the cause of a majority of sporadic cases remains unknown. Recently, epigenetics, especially miRNA studies, show some promising aspects. We aimed to evaluate the differential expression of 10 miRNAs, including miR-9, miR-338, miR-638, miR-663a, miR-124a, miR-143, miR-451a, miR-132, miR-206, and let-7b, for which some connection to ALS was shown previously in ALS culture cells, animal models or patients, and in three miRNA host genes, including *C1orf61* (miR-9), *AATK* (miR-338), and *DNM2* (miR-638), in leukocyte samples of 84 patients with sporadic ALS. We observed significant aberrant dysregulation across our patient cohort for miR-124a, miR-206, miR-9, let-7b, and miR-638. Since we did not use neurological controls we cannot rule out that the revealed differences in expression of investigated miRNAs are specific for ALS. Nevertheless, the group of these five miRNAs is worth of additional research in leukocytes of larger cohorts from different populations in order to verify their potential association to ALS disease. We also detected a significant up-regulation of the *AAKT* gene and down-regulation of the *DNM2* gene, and thus, for the first time, we connected these with sporadic ALS cases. These findings open up new research toward miRNAs as diagnostic biomarkers and epigenetic processes involved in ALS. The detected significant deregulation of *AAKT* and *DNM2* in sporadic ALS also represents an interesting finding. The *DNM2* gene was previously found to be mutated in Charcot-Marie-Tooth neuropathy-type CMT2M and centronuclear myopathy (CNM). In addition, as recent studies connected *AATK* and frontotemporal dementia (FTD) and *DNM2* and hereditary spastic paraplegia (HSP), these two genes together with our results genetically connect, at least in part, five diseases, including FTD, HSP, Charcot-Marie-Tooth (type CMT2M), CNM, and ALS, thus opening future research toward a better understanding of the cell biology involved in these partly overlapping pathologies.

## Introduction

Amyotrophic lateral sclerosis (ALS) is a complex neurodegenerative disease that typically presents in adulthood with symptoms, such as muscle weakness, atrophy and later on paralysis, leading to death within 2–3 years following diagnosis (Rowland and Shneider, 2001). Genetic studies have managed to explain many cases through mutations in several genes for familial ALS (Rosen et al., 1993; Sreedharan et al., 2008; Kwiatkowski et al., 2009; Vance et al., 2009; Dejesus-Hernandez et al., 2011; Renton et al., 2011). However, the cause of a majority of sporadic cases remains unknown, since only ~11% of sporadic ALS is explained by genetic changes (Renton et al., 2011). It is most likely that sporadic disease arises from complex interactions between genetic susceptibility and the environment (Ajroud-Driss and Siddique, 2015).

Sporadic ALS has been linked to many environmental factors, including heavy metal toxicity and exposure to pesticides, fertilizers, smoking, viral infections, physical exercise, and electromagnetic radiation (reviewed in Zufiría et al., 2016). Recent studies show that it is also most likely that epigenetics is a mechanism through which gene-environment interactions are mediated to promote the onset and progression of ALS (Al-Chalabi et al., 2013). Epigenetic mechanisms, including DNA methylation, histone remodeling, RNA editing, and microRNA (miRNA) modifications that are dysregulated in ALS models as well as in ALS patients are probably crucial to understanding ALS pathogenesis, and to establishing a path to treatment (Paez-Colasante et al., 2015).

Therefore, it is necessary to shift the focus to the field of epigenetics, especially miRNAs, which show some promising aspects.

miRNAs are single-stranded non-coding RNA molecules that are ~22 nucleotides in length, which act as post-transcriptional regulators of gene expression either by causing the degradation of target mRNAs or the inhibition of their translation (Pillai et al., 2007). miRNAs are often located within protein coding genes (intragenic miRNA) where they are usually co-transcribed with their host genes. Alternatively, miRNAs can be independent of protein-coding genes (intergenic miRNA) (Bartel, 2004). It has been estimated that miRNAs regulate ~80% of all protein coding genes in mammals (Krol et al., 2010; Huang et al., 2011; Kozomara and Griffiths-Jones, 2014). Many studies have already confirmed the involvement of miRNAs in the development of the nervous system (Conaco et al., 2006; Åkerblom et al., 2012; Zhu et al., 2013). It was concluded that miRNAs are important in many processes taking place in the motor neurons, such as the adequate differentiation of neurons in a certain subtype, maintaining their functionality and regeneration after injuries (Kye and Gonçalves Ido, 2014).

miRNA pathway disruptions could be a cause or consequence of ALS pathology, which is underlain with altered RNA and protein metabolism, cytotoxicity due to faulty glutamate clearance, the inflammatory response, and neuromuscular junction impairments (Paez-Colasante et al., 2015). In addition, in several studies differential expression of miRNAs in ALS have been reported (De Felice et al., 2012; Cloutier et al., 2015; Vrabec et al., 2015; Chen et al., 2016).

In our previous study, we determined genetic changes in 7/84 (8.3%) of Slovenian patients with a sporadic form of ALS (Vrabec et al., 2015). The aim of this study was to evaluate the expression of 10 miRNAs in leukocytes of the same Slovenian sporadic ALS cohort. We selected miR-9, miR-338, miR-638, miR-663a, miR-124a, miR-143, miR-451a, miR-132, miR-206, and let-7b because for each of these miRNAs, some connection to ALS was shown in the preceding studies. Namely, the differential expression of let-7b and miR-663 was detected in TDP-43 knockdown culture cells (Buratti et al., 2010); miR-9 expression was up-regulated in the spinal cord of p.Gly93Ala-SOD1 transgenic mice (Zhou et al., 2013); for miR-132, a role in neurodegeneration in a rat model of ALS was shown (Lungu et al., 2013); the overexpression of miR-206 delays ALS progression and promotes the regeneration of neuromuscular synapses in mice (Williams et al., 2009); miR-638 was dysregulated in the serum of patients with fALS (Freischmidt et al., 2014); the altered miR-124a expression was associated with neuronal fate in p.Gly93Ala-SOD1 ependymal stem progenitor cells (Marcuzzo et al., 2014); miR-143 was dysregulated in the cerebrospinal fluid, serum and lymphoblastoid cell lines of sALS patients (Freischmidt et al., 2013); and miR-338 and miR-451a were differentially expressed in blood leukocytes from patients with sporadic ALS (De Felice et al., 2012, 2014).

In addition, we also focused our attention on investigating the differential expression of three intragenic miRNA host genes, including *C1orf61* (miR-9), *AATK* (miR-338), and *DNM2* (miR-638), which were thus examined, for the first time, in this study in connection with ALS. We selected blood samples as the research material for investigating the miRNA expression for many reasons. The use of human biopsy tissue from skeletal muscle, spinal cord, or frontal cortex is ethically unacceptable in life and is also difficult to obtain from *post-mortem* tissues, while blood samples are easily accessible and represent a non-invasive method for collecting biological material from living patients. In addition, detecting miRNA biomarkers in blood leukocytes may represent the robust biomarkers for the early diagnosis of ALS. Since miRNAs can be easily secreted from neurons and other CNS cells into the extracellular space (Chen et al., 2016) peripheral blood miRNAs expression could be used as indicators in neurological diseases, such as Parkinson's disease (Serafin et al., 2015) and Alzheimer's disease (Cheng et al., 2015).

## Materials and methods

### Patients and control samples

Blood samples of 84 Slovenian patients clinically diagnosed with ALS were collected at the Institute of Clinical Neurophysiology, University Medical Centre Ljubljana, Slovenia. Both genders were represented equally (42 women and 42 men), and none of the patients were related. The mean age of onset was 62 ± 11.72 years (ranging from 37 to 89 years). Sixty-two of the eighty-four (74%) patients had the spinal onset form and 22/84 (26%) had the bulbar onset form of the disease. Four patients (4.8%) had associated symptoms of frontotemporal dementia (FTD), and two patients (2.4%) had some associated symptoms of Alzheimer disease. For 68 patients we obtained information about their treatment. Thirty-one patients were treated with rulizol, and 37 patients were not yet treated. Seven patients had genetic changes (Vrabec et al., 2015), including four patients with a hexanucleotide repeat expansion mutation (HREM) in the *C9ORF72* gene, one patient with a mutation in the *SOD1* gene (p.Val14Met), and one patient with a mutation in the *SOD1* gene (p.Gly93Cys) together with a synonymous alteration c.990A>G (p.Leu330Leu) in *TARDBP*, and in one patient, we detected a synonymous substitution in the *FUS* gene (c.1566G>A, p.Arg522Arg).

As a control group, 27 healthy volunteers were included. There were 14 women and 13 men, with a mean age was 56 years, ranging from 30 to 65 years. The blood samples were collected at the Institute of Clinical Neurophysiology, Division of Neurology, University Medical Centre Ljubljana, Slovenia.

This study was carried out in accordance with the recommendations of the Republic of Slovenia National Medical Ethics Committee with written informed consent from all the subjects. All the subjects gave written informed consent in accordance with the Declaration of Helsinki.

The protocol was approved by the Republic of Slovenia National Medical Ethics Committee.

### RNA isolation

For the RNA isolation, Ficoll-Paque PLUS reagent (Life Sciences, Germany) was used. Briefly, a mixture of blood and PBS buffer (1:2) was carefully layered on Ficoll. A centrifugation step created a gradient and leukocyte layer that was easily transferred into a fresh tube, and the sample was washed with PBS buffer. After washing, the pellet was resuspended in TRI reagent (Sigma-Aldrich, Germany), and further isolation was performed following the manufacturer's protocol. After separation of the water solution containing the RNA, purification was performed by miRNeasy Mini Kit (Qiagen, Germany) following the instructions for purification of total RNA, that includes long and small RNAs as are miRNAs,

### qPCR of miRNA

For purpose of determination of an efficiency of amplification for analyzed miRNAs, initially, pools of the RNA samples from healthy adults and a pool from the ALS patients were created. After reverse transcription and serial cDNA dilution, qPCR efficiency was analyzed for each miRNA (let-7b, miR-9, miR-124a, miR-132, miR-143, miR-206, miR-338, miR-451a, miR-638, and miR-663a) and for each of the three reference genes (*RNU6B, SCARNA17*, and *SNORA73A*) as described below. qPCR was performed in triplicates. The primers used for the expression analysis are listed in Table 1.

**Table 1:** Details of the ready-to-use primers for the expression analyses.

**miRNA/gene**	**Commercial name**	**Catalog number**	**Company**
let-7b	Hs_let-7b_1	MS00003122	miScript, Qiagen
miR-9	Hs_miR-9_1	MS00010752	miScript, Qiagen
miR-124a	Hs_miR-124a_1	MS00006622	miScript, Qiagen
miR-132	Hs_miR-132_1	MS00003458	miScript, Qiagen
miR-143	Hs_miR-143_1	MS00003514	miScript, Qiagen
miR-206	Hs_miR-206_1	MS00003787	miScript, Qiagen
miR-338	Hs_miR-338_1	MS00003990	miScript, Qiagen
miR-451a	Hs_miR-451_1	MS00004242	miScript, Qiagen
miR-638	Hs_miR-638_4	MS00043624	miScript, Qiagen
miR-663a	Hs_miR-663_3	MS00037247	miScript, Qiagen
RNU6B	Hs_RNU6-2_11	MS00033740	miScript, Qiagen
SCARNA17	Hs_SCARNA17_11	MS00014014	miScript, Qiagen
SNORA73A	Hs_SNORA73A_11	MS00014021	miScript, Qiagen
AATK	Hs_AATK_1_SG	QT01160264	QuantiTect, Qiagen
C1orf61	Hs_C1orf61_1_SG	QT01014790	QuantiTect, Qiagen
DNM2	Hs_DNM2_1_SG	QT00037072	QuantiTect, Qiagen
GAPDH	Hs_GAPDH_1_SG	QT00079247	QuantiTect, Qiagen
U6	Hs_USB1_1_SG	QT00066906	QuantiTect, Qiagen

Prior to qPCR, reverse transcription of 100 ng of RNA was performed using the miScript II Reverse Transcription Kit (Qiagen, Germany) with 5x miScript HiFlex buffer which enables transcription of mRNA as well as miRNA to cDNA. Inhibitor RNase (Qiagen, Germany) was also added to the reaction mixture. Reverse transcription was performed in a total volume of 10 μl according to the manufacturer's instructions.

A miScript Sybr Green PCR Kit (Qiagen, Germany) was used for all the qPCR reactions according to manufacturer's protocol in a 10 μl reaction volume. Specific primers for the miRNAs and reference genes and the 10x miScript Primer Assay (Qiagen, Germany) were used. Based on the efficiency analysis, all the ALS and control cDNA samples were diluted 1:100, and the reactions were performed on a Rotor Gene Q (Qiagen, Germany) in duplicate for each of 111 sample.

Information about the miRNA location (intragenic or intergenic) was gained through the online tools miRIAD (http://www.bioinfo.mochsl.org.br/miriad) and miRBase (http://www.mirbase.org/), and further host genes for the intragenic miRNA were determined.

### qPCR of intragenic miRNA's host genes

qPCR was also used to identify host gene expression. For qPCR, the cDNA from miScript System was used, that was resulted from reverse transcription with HiFlex buffer, which enables reverse transcription also of mRNA using oligo-dT primers. Three genes, *C1orf61* (miR-9), *AATK* (miR-338), and *DNM2* (miR-638), were analyzed. Sybr Select Master Mix (Life Technologies, USA) was used for all the reactions according to the manufacturer's instructions in a 10 μl reaction volume. Specific primers with the 10x QuantiTect Primer Assay (Qiagen, Germany) were added to the reaction mixture. *GAPDH* and *U6* were analyzed as reference genes. All the remaining protocols are the same as described in section qPCR of miRNA *qPCR and statistical analysis of miRNA*. The primers used for the expression analysis are listed in Table 1.

### Statistical analysis

To analyze qPCR data from miRNA and mRNA expression analysis, efficiency corrected model of 2^−Δ*ΔCt*^ was used (Latham, 2010). Briefly, based on the results of efficiency, whenever necessary, Cts were recalculated. Then the resulting Cts were used for calculation of ΔCt between GOI (gene of interest) and geometric mean of RGs (reference genes) for each individual ALS sample and control sample. The calculated ΔCts of the ALS patients and control group were tested for statistical significance with the statistical software SPSS ver. 20 (SPSS Inc., USA) using a Mann–Whitney test. Within the group of ALS patients, the ΔCts were, in addition, analyzed according to gender, disease onset and whether or not the mutation was previously detected. Within the control group, the ΔCts were also analyzed according to gender. A Spearmans' correlation between the expression of miRNAs and their host genes was also calculated. All the correlations that were below the cut-off of *p* < 0.05 were treated as significant. Non-parametric methods were used since there was non-normal distribution of certain Cts and ΔCts within both groups of samples, sALS, and controls.

### Pathway analysis of investigated miRNAs

Using Human MicroRNA Disease Database (Lu et al., 2008; Li et al., 2014) we have search for validated gene targets of investigated miRNAs in humans. We also used mirPath database (Vlachos et al., 2015) which can utilize experimentally validated miRNA interactions derived from DIANA- TarBase v6. in order to investigate in which pathways miRNAs of this study are already determined to be involved (Vlachos et al., 2015).

## Results

### miRNA expression in leukocytes from sALS patients and statistical analysis

**Figure 1:**
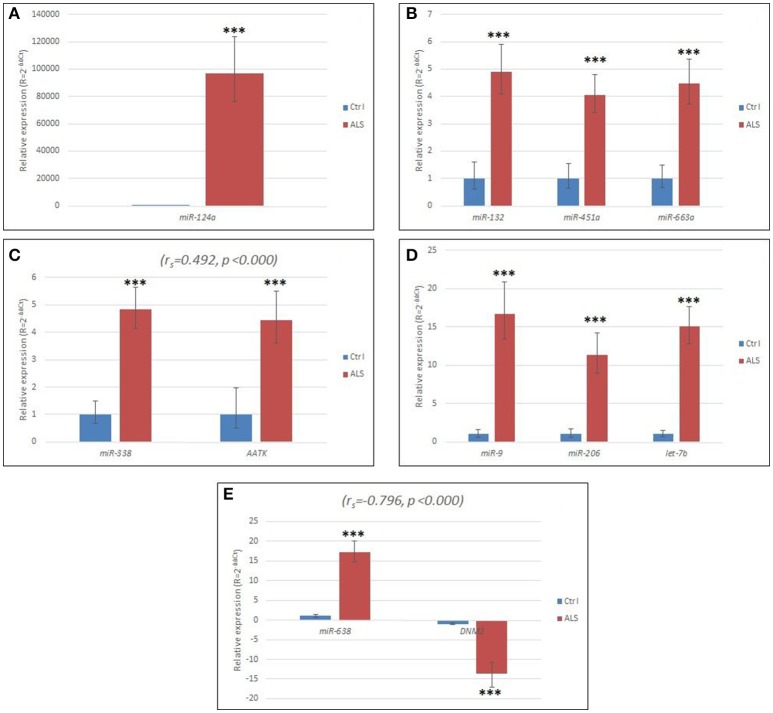
**(A)** Relative expression of miR-124 in sALS patients compared to controls. **(B)** Relative expression of miR-132, miR-451a, and miR-663a, respectively, in sALS patients compared to controls. **(C)** Relative expression of miR-338 and its host gene *AATK* in sALS patients compared to controls. **(D)** Relative expression of miR-9, miR-206, and let-7b, respectively in sALS patients compared to controls. **(E)** Relative expression of miR-638 and its host gene *DNM2* in sALS patients compared to controls. Ctrl, control group; ΔCt, delta Ct; ΔΔCt, delta delta Ct; sALS, sporadic amyotrophic lateral sclerosis; ^***^p < 0.000. Horizontal lines represent standard deviation.

The detailed relative expressions of each miRNA analyzed are presented as the average relative expressions in the graphs (Figures 1A–E) and as the ΔCt in Table 2.

**Table 2:** ΔCt of analyzed miRNAs and two host genes.

	**ALS**			**Ctrl**		
**miRNA/gene**	**ΔCt_min_**	**ΔCt_max_**	**Median ΔCt**	**ΔCt_min_**	**ΔCt_max_**	**Median ΔCt**
let-7b	0.65	3.79	2.32	3.68	8.69	5.93
miR-9	7.13	10.9	8.95	9.27	19.72	12.74
miR-124a	7.69	10.11	8.82	21.40	28.93	24.50
miR-132	5.66	8.69	6.68	6.33	12.19	8.85
miR-143	5.39	13.81	10.12	5.96	13.73	9.84
miR-206	10.60	16.39	11.65	12.59	18.66	15.94
miR-338	3.82	9.50	7.98	4.41	14.38	9.90
miR-451a	−1.00	5.23	2.43	1.34	8.26	4.46
miR-638	1.16	4.05	2.76	4.76	9.02	6.65
miR-663a	1.47	3.48	2.38	2.10	6.81	4.36
DNM2	6.97	10.56	8.76	1.63	7.32	4.86
AATK	6.08	11.25	9.47	−0.89	15.50	12.07

*ALS, amyotrophic lateral sclerosis patients; Ctrl, controls; Ct, threshold cycle; ΔCt, delta Ct; ΔCt_min_, minimal ΔCt detected within a group; ΔCt_max_, maximal ΔCt detected within a group; median ΔCt, median of all individual detected ΔCts between maximal and minimal ΔCts within the group of patients/controls*.

We found that all the miRNAs were significantly up-regulated (*p* < 0.001), except for miR-143, which was expressed at a similar level in the ALS patients and in the control group. We found that miR-124 was highly elevated in all the sALS patients (Figure 1A, Table 2). The relative up-regulation of four miRNAs, miR-451a, miR-132, miR-338, and miR-663a, was similar for the sALS group compared to the control group and ranged from 4.06- to 4.90-fold (Figures 1B,C), whereas the relative up-regulation of miR-9, miR-206, miR-638, and let-7b ranged from 11.32- to 17.19-fold (Figures 1D,E).

The expression changes were statistically evaluated according to gender, the presence of mutations and disease onset, but no significant differences were detected. We only found significant differences in the expression of miR-143 by gender (*p* = 0.001), but the difference in expression was also observed in the control group (*p* = 0.029).

### Expression of the miRNA host genes C1orf61, AATK, and DNM2

The *C1orf61* (miR-9) gene was not expressed in the blood samples of the ALS patients or in the blood samples of the control RNAs. The *AATK* and *DNM2* host genes of miR-338 and miR-638, respectively, were statistically significant up- and down-regulated (*p* < 0.001 for both), respectively, in the leukocytes for the sALS patients, and their relative expression is shown in Figures 1C,E. The host gene expression changes were statistically evaluated the same way as in the case of the miRNAs, i.e., according to gender, the presence of mutations and disease onset, but no significant differences were detected. The ΔCts are listed in Table 2.

### Comparison of rulizol treated and untreated patients

Using Mann–Whitney test, we have compared ΔCt of patients that were treated using rulizol, to ΔCt of patients that were not yet treated. For five miRNAs (miR-143, miR-451, miR-338, miR-638, let-7b) and for two host genes (*AATK, DNM2*), we did not observe significant difference in expression between treated and untreated patients. However, we found relatively slight but statistically significant down-regulation of miR-124a (1.2-fold, *p* = 0.043), miR-132 (1.5-fold, *p* = 0.002) miR-206 (4.4-fold, *p* = 0.001), and miR-663a (1.3-fold, *p* = 0.019), whereas miR-9 showed up-regulation (1.8-fold, *p* = 0.045) (data not shown). However, all these miRNAs still remained significantly up-regulated when compared to healthy adult control group regardless on the application of treatment.

### Correlation between the miRNAs and their host genes

We observed a moderate positive correlation between miR-338 and its host gene *AATK* (ρ = 0.482, *p* < 0.001) and also moderate negative correlation between miR-638 and its host gene *DNM2* (ρ = −0.519, *p* < 0.001). Results are presented in Figures 1C,E. Interestingly, within the control group, there was no correlation between the expression of miR-338 and its host gene *AATK*. However, even more interestingly, miR-638 and its host gene *DNM2* were expressed with a moderate positive correlation (ρ = 0.442, *p* = 0.021).

### Pathway analysis of investigated miRNAs

**Figure 2:**
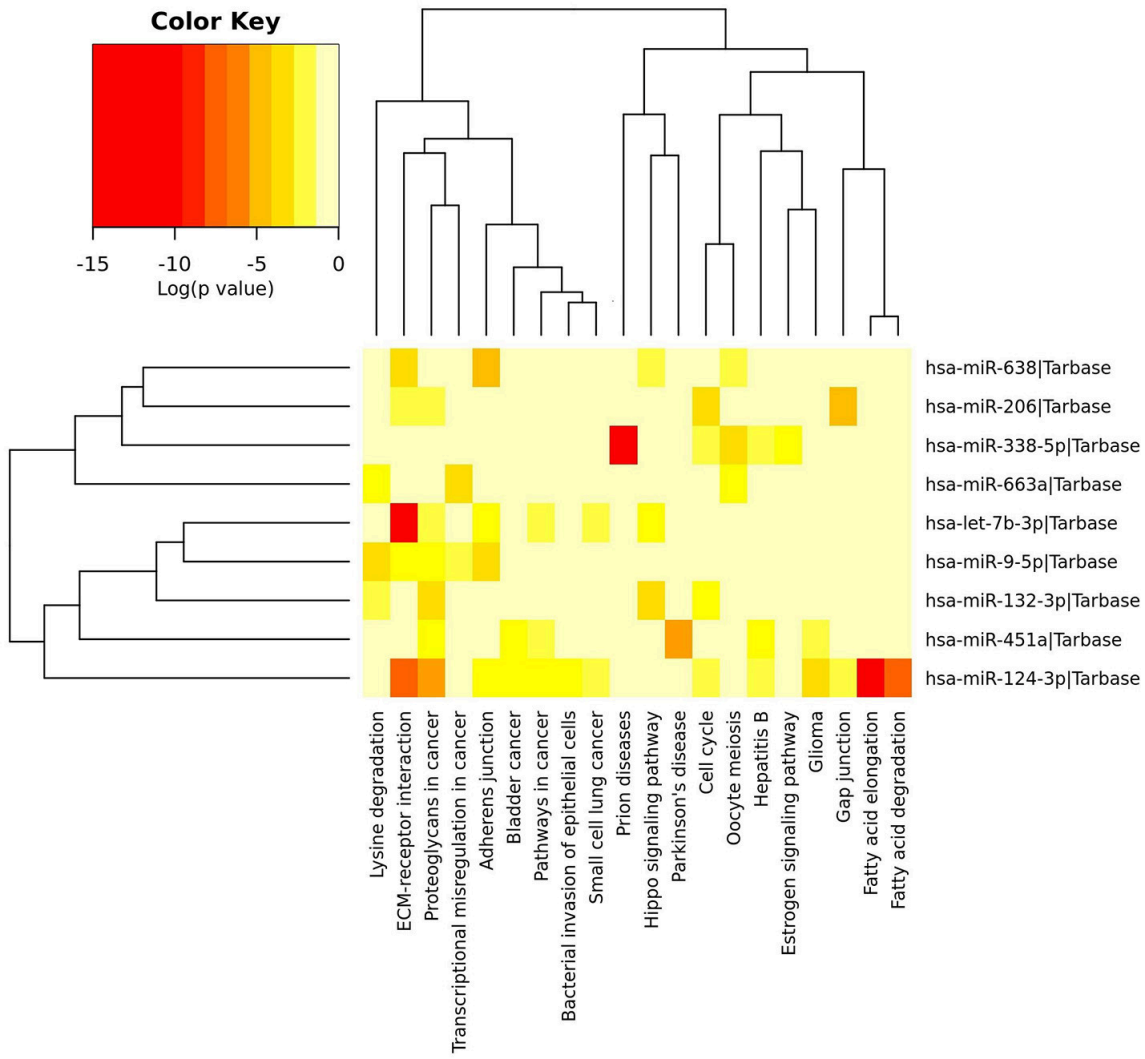
Heatmap of experimentally validated union pathways of investigated group of miRNAs using miRPath Database (Vlachos et al., 2015). In union pathways the enrichment analysis is performed and significance levels (*p*-values) are calculated between each miRNA and every pathway. Then, for each pathway a merged *p*-value is extracted using Fisher's meta-analysis method, which signify if a particular pathway is targeted by at least one miRNA out of the initially selected group (Vlachos et al., 2015). Different colors of cells mean different level of significance [log (*p*-value) in Color Key].

Using Human MicroRNA Disease Database we found that for majority of miRNAs investigated in this study there is at least one confirmed target gene in humans. For two miRNAs, namely miR-638 and miR-663a, there have been no target yet confirmed. However, the majority of confirmed target genes have been investigated in different neoplasms and none in ALS. Results are summarized in Table 3. Figure 2 represents heatmap of union pathways of investigated group of miRNAs derived from experimentally validated data using miRPath v.3 DIANA-TarBase, Database (Vlachos et al., 2015; Paraskevopoulou et al., 2016).

**Table 3:** List of validated target genes of investigated miRNAs according to Human MicroRNA Disease Database (Lu et al., 2008; Li et al., 2014).

**miRNA**	**Target genes**	**Diseases**
miR-9	ITGB1	Breast cancer
	JAK, CAMTA1	Glioblastoma
	MMP14	Neuroblastoma
	ETS1, NKFB1, CDX2, CCND1	Gastric cancer
miR-124a	IQGAP1	Breast cancer
	ROCK2, EZH2, PIK3CA	Hepatocellular carcinoma
	CDK4	Glioma
	CDK6, HMGA1	Medulloblastoma
	AR	Prostatic cancer
miR-132	TMEM106B	Dementia
miR-143	GCK, MACC1, DNMT3A, KRAS	Colorectal cancer
	ERK5	B-cellular lymphoma, Obesity
	RAS	Pancreatic cancer
	PTGS2, SERPINE1, BCL2	Uterine cervical neoplasm
miR-206	ESR1	Breast cancer
	Notch3	Neoplasm
	MET	Rhabdomyosarcoma
	CCND2	Gastric cancer
miR-338	SMO	Hepatocellular carcinoma
	CCND1	Hepatitis B
miR-451a	IKBKB	Hepatocellular carcinoma
	RAB14	Non-small cell lung cancer
	YWHAZ	Diabetic nephropathy
	MDR, ABCB1	Neoplasm
miR-638	/	
miR-663a	/	
let-7b	BCL2L1	Hepatocellular carcinoma
	KRAS	Non-small cell lung cancer
	IL13	Inflammation
	ITGB3, CCND1	Melanoma
	HMGA2, RAS	Neoplasm

## Discussion

Any novel findings contribute to the understanding of ALS pathogenesis. Functional studies show that miRNAs are involved in virtually all the cellular processes investigated and that the changes in their expression are closely related to the occurrence of the disease (Filipowicz et al., 2008). In addition, a list of miRNAs detected to be dysregulated in the CNS and the periphery is growing in mouse models and patients with various neurodegenerative diseases (Goodall et al., 2013). Specifically, in ALS patients, dysregulated miRNAs have been detected in the spinal cord and serum as well as in leukocytes (Butovsky et al., 2012; De Felice et al., 2012, 2014; Campos-Melo et al., 2013; Freischmidt et al., 2014, 2015). In the context of ALS, alterations in the abundance of peripheral miRNAs have been suggested to represent a systemic dysfunction of ubiquitously expressed RNA-binding proteins that are involved in the pathogenesis of ALS (Freischmidt et al., 2013). miRNAs are detected in serum, plasma and other body fluids in a remarkably stable form, as exosomal cargos or bound to specific proteins, making them attractive biomarkers for human diseases (Xu et al., 2012). In this study, we determined the differential expression of 10 miRNAs, including miR-9, miR-338, miR-638, miR-663a, miR-124a, miR-143, miR-451a, miR-132, miR-206, and let-7b, in blood leukocytes. At the beginning of our study, the expression of seven (let-7b, miR-124a, miR-132, miR-206, miR-663a, miR-9, and miR-638) out of ten studied miRNAs was not previously examined in blood leukocytes of ALS patients. In addition, the dysregulated expression of the host genes *DNM2* (miR-638) and *AATK* (miR-338) has been demonstrated for the first time in connection with ALS in this study.

To understand the pathogenesis of ALS, it is crucial to be familiar with the signaling that occurs between the fibers of the skeletal muscles and motor neurons (Kovanda et al., 2014). In this signaling, muscle-specific miR-206 is involved. An elevated expression of miR-206 was shown in a mouse model of ALS, and its under-expression is expected to accelerate the progression of the disease (Williams et al., 2009). An increased expression of miR-206 was also shown in samples of skeletal muscle of human patients with ALS (Russell et al., 2013). Toivonen et al. studied alterations of several miRNAs from the skeletal muscle and plasma of SOD1-p.Gly93Ala mice and, subsequently, tested the levels of the affected miRNAs in the serum from human ALS patients. In animal models age/gender/muscle groups, miR-206 was the only one consistently altered during the course of the disease pathology. Mature miR-206 was increased in fast-twitch muscles preferably affected in the SOD1-p.Gly93Ala model in both sexes, with highest expression in most severely affected animals. miR-206 was also increased in the circulation of symptomatic animals and in a group of 12 definite ALS patients tested. They concluded that miR-206 is elevated in the circulation of symptomatic SOD1-p.Gly93Ala mice and possibly in human ALS patients (Toivonen et al., 2014). An increased expression of this miRNA in the serum of sALS patients compared to controls was recently identified (Waller et al., 2017). In our study, we further confirmed the involvement of miR-206 in the circulation in human ALS, since in our cohort of ALS patients, we demonstrated a significant increased expression of miR-206 in the peripheral blood leukocytes. The question, however, still exists whether the elevated expression of miR-206 is the result of the disease or its cause.

The exosomal-mediated transfer of miRNAs is possible and was determined for miR-124a (Morel et al., 2013) and miR-638 (Goldie et al., 2014). Morel et al. (2013) revealed the exosome-mediated transfer of miR-124a in a neuron-to-astrocyte communication pathway. They demonstrated that miR-124a was selectively reduced in the spinal cord tissue of end-stage SOD1-p.Gly93Ala mice. Subsequent exogenous delivery of miR-124a *in vivo* through into spinal cord of ALS mice significantly prevented further pathological loss and led to a 30% increase in the expression of the excitatory amino acid transporter 2 (EAAT2, rodent analog GLT1), which is responsible for the uptake of glutamate from the synaptic cleft (Morel et al., 2013). In a recent publication (Marcuzzo et al., 2015), the expression levels of miR-124a in the brain of p.Gly93Ala-SOD1 mice at late-stage disease (week 18) were significantly higher relative to Wt-SOD1 mice, whereas interestingly, the relative expression levels of this miRNA were significantly lower in the whole spinal cord of the p.Gly93Ala-SOD1 mice than in the whole spinal cord of the Wt-SOD1 mice. However, in the recently published study of Cunha et al., the spinal cord of symptomatic stage p.Gly93Ala-SOD1 mice showed an up-regulation of miR-124a (Cunha et al., 2017).

In the leukocytes of a Chinese sALS cohort, Chen et al. identified significantly lower expression levels of several miRNAs, including hsa-miR-124, in comparison with healthy controls using a microarray (Chen et al., 2016).

The relative expression levels of miR-124a in the peripheral blood leukocytes of sALS patients was also studied. We detected a significantly elevated expression of miR-124a in all 84 sALS samples compared to the controls regardless of the onset of the disease. Since the differential expression of miR-124a was shown in the brain and in the spinal cord of the ALS mice as well as in the leukocytes of patients with sALS, this might indicate the possible relationship between CNS and peripheral tissues and places miR-124a among the miRNAs that are worthy additional investigations in the direction toward ALS disease biomarkers and therapeutic targets, especially since an emerging critical role of microglia and astrocytes has been established in the etiology of ALS (Radford et al., 2015).

Our results showed an elevated relative expression of let-7b in the leukocytes of the Slovenian sALS patients studied. The dysregulation of let-7b was, until now, determined in connection with ALS in TDP-43 knockdown in culture cells, where the authors showed that the removal of TDP-43 from the cell nucleus caused specific downregulation of let-7b that could further influence the expression of other potential transcripts involved in neurodegeneration and synapse formation (Buratti et al., 2010).

In a study by Freischmidt et al. they observed significant differences in the relative levels of let-7b, miR-143 and miR-132 in the serum of sALS patients compared to the mean expression of the healthy controls. They found that the mean relative expression of all three miRNAs significantly decreased in their sALS cohort (Freischmidt et al., 2013). However, Waller et al. recently reported the up-regulation of miR-143-3p in the serum of sALS (Waller et al., 2017). In this study, we found almost no difference in the mean relative expression of miR-143-3p in leukocytes, which was similar in the sALS patients and in the controls. In addition, we found an up-regulated relative expression of miR-132 in the leukocytes. A significant increase in miR-132 was previously reported in affected rats, where it was also demonstrated that miR-132 regulates Nurr1 levels and, thus, might influence the development and function of midbrain dopaminergic neurons (Lungu et al., 2013).

We observed the differential expression of intergenic miR-451a and miR-663a. Using a microarray strategy, De Felice et al. evaluated the expression of miRNAs in the leukocytes of 8 sALS patients and 12 unaffected healthy controls. They identified seven miRNAs, including miR-451a, that were down-regulated across different gender groups and in all the tested sALS samples (De Felice et al., 2012). In our study, we observed a relative up-regulation of miR-451a in our patient samples. Until now, miR-451a was shown to be involved in several cancers, including glioma and gastric cancer, where it acts as a potential tumor suppressor affecting cell proliferation, invasion and apoptosis, perhaps via the regulation of the PI3K/AKT /mTOR signaling pathway (Nan et al., 2010; Riquelme et al., 2015). Recently, Chen et al. reported the identification of four under-expressed microRNAs in the leukocytes of Chinese patients, including hsa-miR-451, having a high diagnostic accuracy of sALS (Chen et al., 2016). The functional role of miR-451a in ALS pathology still needs to be revealed.

Changes in the expression of miR-663a have been studied following TDP-43 knockdown in culture cells, where miR-663a up-regulation was observed (Buratti et al., 2010). We detected a significant relative up-regulated miR-663a in the patient leukocyte samples compared to the control samples. miR-663a is dysregulated in connection with other diseases, such as pancreatic cancer (Lin et al., 2014) and autism (Mundalil Vasu et al., 2014).

Three of the investigated miRNAs, miR-9, miR-338-3p, and miR-638, were intragenic. The expression of miR-9 was previously studied in p.Gly93Ala-SOD1 mice, and its significant increase was detected in whole brain at late stage disease compared to Wt-SOD1 control brains as well as in manually dissected brainstem motor nuclei and primary motor cortex (Marcuzzo et al., 2015). On the other hand, Zhang et al. reported the down-regulation of miR-9 in induced pluripotent stem cell-derived neurons of FTD/ALS patients with TDP-43 mutations (Zhang et al., 2013). We found that the mean miR-9 leukocyte expression was up-regulated in sALS patients compared to controls. miR-9 is highly expressed in the nervous system (Lagos-Quintana et al., 2002) and is an important factor regulating neurogenesis. Its role is to manage the growth of axons through the regulation of mRNA translation protein MAP1B (Dajas-Bailador et al., 2012). A change in the expression of miR-9 during the development of motor neurons leads to a modified subtype of motor neurons in chicken embryos (Otaegi et al., 2011a). In this model, miR-9 indirectly affects the development and differentiation of neurons in the spinal cord (Otaegi et al., 2011b).

De Felice et al. reported over-expression of miR-338-3p in blood leukocytes as well as in cerebrospinal fluid, serum, and spinal cord from sALS patients (De Felice et al., 2014). We also found significantly over-expressed miR-338-3p in the leukocyte samples of Slovenian sALS patients. Several studies have already shown that miR-338-3p has functions in controlling several different molecular pathways. The expression of miR-338-3p was significantly down-regulated in colorectal carcinoma (Xue et al., 2014) and in esophageal squamous cell carcinoma (Yang et al., 2013), and it is demonstrated that miR-338-3p functions as a tumor suppressor in human non-small-cell lung carcinoma where it targets Ras-related protein 14 (Sun et al., 2015). Using deep sequencing of the plasma fraction enriched in exosomes, Lugli et al. showed the decreased expression of seven miRNAs, including miR-338-3p, in samples of patients with Alzheimer disease (Lugli et al., 2015). Through gain and loss of function experiments *in vitro* and *in vivo*, it was demonstrated that miR-338 positively regulates oligodendrocyte differentiation by directly targeting genes such as Hes5 and Sox6 and inhibiting genes involved in neuronal differentiation (Zhao et al., 2010).

miR-638 was up-regulated in all 84 samples in our sALS cohort. In one previous study, leukocytes from 8 patients with sporadic ALS and from 12 unaffected healthy controls were analyzed using a microarray analysis. The differential expression of miR-638 was detected and was down-regulated in eight analyzed sALS samples (De Felice et al., 2012). In two other studies, Freischmidt et al. detected the reduced expression of miR-638 in the serum of patients with a hereditary form of ALS (Freischmidt et al., 2014) and a heterogeneous relative dysregulation (down- or up-regulation) in the serum of patients with the sporadic form of ALS (Freischmidt et al., 2015). The role of miR-638 in connection with ALS is not yet reported. Several functions of miR-638 have been associated with the suppression or progression of various cancers (Tay et al., 2014; Zhang et al., 2014; Lin et al., 2015; Wang et al., 2015). In addition, Goldie et al. investigated the subcellular distribution of miRNAs in resting and potassium chloride depolarized human neuroblasts. They found both selective enrichment and depletion of miR-638 in neurites. Their findings support a role for miR-638 as regulator of neural plasticity, as it is compartmentalized in neurons and undergo activity-associated redistribution or release into the extracellular matrix (Goldie et al., 2014).

To further elucidate the functional role of investigated miRNAs we performed *in-silico* analyses using publically available databases (Lu et al., 2008; Li et al., 2014; Vlachos et al., 2015; Paraskevopoulou et al., 2016). Majority of experimentally validated gene targets of in this study investigated miRNAs' have been by now connected to various neoplasms and cancers (Table 3). Similar results have been revealed when constructing Heatmap between biological pathways/categories and a group of significantly deregulated miRNAs of this study (Figure 2) using experimentally validate data from miRPath v.3 DIANA-TarBase, database (Vlachos et al., 2015; Paraskevopoulou et al., 2016). Namely, for 7 out of 9 miRNAs the involvement in pathways to cancers or different forms of cancer was detected (Figure 2—Proteoglycans in cancer pathway, Transcriptional misregulation in cancer pathway, Bladder cancer, Small cell lung cancer, Glioma). The fact that each miRNA can regulate hundreds of different targets and that cancer is the most investigated disease, the outcome results are not surprising. Two miRNAs were detected in significant connection with neurodegenerative diseases, respectively, miR-451a with Parkinson's disease and miR-338-5p with prion diseases. Since we did not use neurological controls in our study we can't rule out that revealed differences in expression of investigated miRNAs are specific for ALS, although the use of a group of several miRNAs simultaneously may improve the specificity.

The differential expression of three miRNA host genes, including *C1orf61* (miR-9), *AATK* (miR-338), and *DNM2* (miR-638), were studied, for the first time, in connection with ALS. We did not detect *C1orf61* expression in the leukocyte samples of the ALS patients nor in the leukocyte samples of the controls. *C1orf61* is a brain-specific transcriptional activator of c-fos, which is expressed from proliferation through maturation of multiple neuronal cell types (Jeffrey et al., 2000). However, we detected an elevated relative expression of *AATK* and a reduced relative expression of *DNM2* in the tested sALS leukocytes samples compared to the control samples, and thus, for the first time, we connected these two genes with ALS.

The protein encoded by the *AATK* gene is apoptosis-associated tyrosine kinase, which is induced during apoptosis. Expression of this gene may be necessary for the induction of growth arrest and/or apoptosis of myeloid precursor cells. It has been shown in a neuroblastoma cell-line that *AATK* gene produce neuronal differentiation (Raghunath et al., 2000). Tomomura et al. analyzed the expression profiles of *AATK* (also known as AATYK) in developing mouse brains. Their results suggest the role of *AATK* in cell death in mature neurons, as well as unique role in promoting neurite extension through its tyrosine kinase activity in developing neurons (Tomomura et al., 2003).

It was shown that Cdk5-LMTK1-Rab11A pathway is a regulatory mechanism of dendrite development and axon outgrowth (Takano et al., 2014). In addition, recently, Ferrari et al. performed a case-control association study in large Italian FTD cohort (*n* = 530). They identified 2 novel potential loci for frontotemporal dementia (FTD). Suggestive alleles at 17q25.3 identified a disease-associated haplotype causing decreased expression of -cis genes such as *RFNG* and *AATK* involved in neuronal genesis and differentiation and axon outgrowth, respectively (Ferrari et al., 2015).

The significant up-regulation of *AATK* in the leukocyte samples detected in our cohort of sALS patients might, thus, further suggest both the possible connection of *AATK* and ALS and the additional confirmation of the genetic overlap between ALS and FTD.

It is also known that ALS shares genetic characteristics with other complex diseases, including Charcot-Marie-Tooth (CMT4J); namely, mutations in *FIG4*, a phosphatase that regulates intracellular vesicle trafficking along the endosomal-lysosomal pathway, have been associated with both diseases (Kon et al., 2014). Interestingly, the *DNM2* gene, which we revealed as down-regulated in our sALS patient samples, was also previously found to be mutated in Charcot-Marie-Tooth neuropathy type CMT2M, a motor and sensory neuropathy primarily affecting peripheral nerves (Züchner et al., 2005) and in centronuclear myopathy (CNM), presenting with primary damage in skeletal muscles (Bitoun et al., 2005). The ubiquitously expressed large GTPase Dynamin 2 (DNM2) plays a critical role in the regulation of intracellular membrane trafficking through its crucial function in membrane fission, particularly in endocytosis. Tinelli et al. examined the consequences of DNM2 loss in skeletal muscle cells. They found that loss of DNM2 function in skeletal muscles initiates a chain of harmful parallel and serial events, involving dysregulation of lipid droplets and mitochondrial defects within altered muscle fibers, defective neuromuscular junctions and peripheral nerve degeneration (Tinelli et al., 2013).

Furthermore, hereditary spastic paraplegia (HSP) is another complex disease that exhibits some genetic overlap with ALS through mutations in *ALS2* and *SPG11* (Su et al., 2014). Recently, whole exome sequencing identified candidate genetic variants, a missense c.2155C > T, p.Arg719Trp mutation in the highly conserved GTP-effector domain of *DNM2* in four-generation Siberian kindred showing clinical features of HSP (Sambuughin et al., 2015).

Thus, dysfunctional or dysregulated *DNM2* connects hereditary spastic paraplegia, Charcot-Marie-Tooth neuropathy (CMT2M), and centronuclear myopathy (CNM), and according to our new findings, it also connects at least a subset of sporadic ALS cases.

## Conclusions

In conclusion, we detected the differential expression of 10 miRNAs involved in the ALS pathology in the leukocyte samples of patients affected with the sporadic form of ALS. Seven of these miRNAs have not been previously investigated in peripheral blood leukocytes. We observed significant aberrant dysregulation across our patient cohort for miR-124a, miR-206, miR-9, let-7b, and miR-638. Since we did not use neurological controls in this study we cannot conclude that the revealed differences in expression of investigated miRNAs are specific for ALS. Nevertheless, the group of these five miRNAs is worth of additional research in leukocytes of larger cohorts from different populations in order to verify their potential association to ALS disease. The detected significant up-regulation of *AAKT* and down-regulation of *DNM2* in sporadic ALS represents an exciting new finding. However, it needs additional research. Since the connection between *AATK* and frontotemporal dementia and *DNM2* and Charcot-Marie-Tooth (type CMT2M), centronuclear myopathy (CNM), and hereditary spastic paraplegia was discovered in recent studies, these two genes together with the results in this study genetically connect, at least in part, five diseases, including FTD, HSP, CMT2M, CNM, and ALS, and thus opens future research toward a better understanding of the cell biological processes involved in these partly overlapping complex clinical syndromes.

## Author contributions

MR-G: Substantially contributed to the conception and design of the work and wrote the paper; KV and EB: Substantially contributed to the acquisition, analysis, and interpretation of the data; BK, LD, LL, and JZ: Substantially contributed to the acquisition and interpretation of the data; BR and DG: Substantially contributed to the conception and design of the study. All the authors contributed in critically revising the manuscript for important intellectual content and gave the final approval of the version to be published. All the authors agree to be accountable for the content of the work.

### Conflict of interest statement

The authors declare that the research was conducted in the absence of any commercial or financial relationships that could be construed as a potential conflict of interest.
